# Multi-parametric and multi-regional histogram analysis of MRI: modality integration reveals imaging phenotypes of glioblastoma

**DOI:** 10.1007/s00330-018-5984-z

**Published:** 2019-02-01

**Authors:** Chao Li, Shuo Wang, Angela Serra, Turid Torheim, Jiun-Lin Yan, Natalie R. Boonzaier, Yuan Huang, Tomasz Matys, Mary A. McLean, Florian Markowetz, Stephen J. Price

**Affiliations:** 1 Cambridge Brain Tumour Imaging Laboratory, Division of Neurosurgery, Department of Clinical Neurosciences, University of Cambridge, Box 167 Cambridge Biomedical Campus, Cambridge, CB2 0QQ UK; 2 Department of Neurosurgery, Shanghai General Hospital (originally named “Shanghai First People’s Hospital”), Shanghai Jiao Tong University School of Medicine, Shanghai, China; 3 The Centre for Mathematical Imaging in Healthcare, Department of Pure Mathematics and Mathematical Statistics, University of Cambridge, Cambridge, UK; 4 Department of Radiology, University of Cambridge, Cambridge, UK; 5 Faculty of Medicine and Health Technology, Tampere University, Tampere, Finland; 6 Institute of Biosciences and Medical Technologies (BioMediTech), Tampere, Finland; 7 NeuRoNe Lab, DISA-MIS, University of Salerno, Fisciano, SA Italy; 8 Cancer Research UK Cambridge Institute, University of Cambridge, Cambridge, UK; 9 CRUK & EPSRC Cancer Imaging Centre in Cambridge and Manchester, Cambridge, UK; 10 Department of Neurosurgery, Chang Gung Memorial Hospital, Keelung, Taiwan; 11 Chang Gung University College of Medicine, Taoyuan, Taiwan; 12 Developmental Imaging and Biophysics Section, Great Ormond Street Institute of Child Health, University College London, London, UK; 13 Wolfson Brain Imaging Centre, Department of Clinical Neurosciences, University of Cambridge, Cambridge, UK

**Keywords:** Glioblastoma, Magnetic resonance imaging, Machine learning, Survival analysis, Prognosis

## Abstract

**Objectives:**

Integrating multiple imaging modalities is crucial for MRI data interpretation. The purpose of this study is to determine whether a previously proposed multi-view approach can effectively integrate the histogram features from multi-parametric MRI and whether the selected features can offer incremental prognostic values over clinical variables.

**Methods:**

Eighty newly-diagnosed glioblastoma patients underwent surgery and chemoradiotherapy. Histogram features of diffusion and perfusion imaging were extracted from contrast-enhancing (CE) and non-enhancing (NE) regions independently. An unsupervised patient clustering was performed by the multi-view approach. Kaplan-Meier and Cox proportional hazards regression analyses were performed to evaluate the relevance of patient clustering to survival. The metabolic signatures of patient clusters were compared using multi-voxel spectroscopy analysis. The prognostic values of histogram features were evaluated by survival and ROC curve analyses.

**Results:**

Two patient clusters were generated, consisting of 53 and 27 patients respectively. Cluster 2 demonstrated better overall survival (OS) (*p* = 0.007) and progression-free survival (PFS) (*p* < 0.001) than Cluster 1. Cluster 2 displayed lower N-acetylaspartate/creatine ratio in NE region (*p* = 0.040). A higher mean value of anisotropic diffusion in NE region was associated with worse OS (hazard ratio [HR] = 1.40, *p* = 0.020) and PFS (HR = 1.36, *p* = 0.031). The seven features selected by this approach showed significantly incremental value in predicting 12-month OS (*p* = 0.020) and PFS (*p* = 0.022).

**Conclusions:**

The multi-view clustering method can provide an effective integration of multi-parametric MRI. The histogram features selected may be used as potential prognostic markers.

**Key Points:**

*• Multi-parametric magnetic resonance imaging captures multi-faceted tumor physiology.*

*• Contrast-enhancing and non-enhancing tumor regions represent different tumor components with distinct clinical relevance.*

*• Multi-view data analysis offers a method which can effectively select and integrate multi-parametric and multi-regional imaging features.*

**Electronic supplementary material:**

The online version of this article (10.1007/s00330-018-5984-z) contains supplementary material, which is available to authorized users.

## Introduction

Glioblastoma represents the most common primary brain malignancy in adults, characterized by dismal prognosis [1]. The remarkable heterogeneity of glioblastoma may cause inconsistent treatment response among patients. Despite many molecular markers having been identified to be of prognostic and/or diagnostic value, imaging markers provide crucial pre-treatment information for patient management. There is a pressing but unmet need for validated imaging markers to assess interpatient variability, plan personalized treatment, and predict response.

MRI shows potential in evaluating glioblastoma heterogeneity non-invasively. Although widely used in clinical practice, structural imaging is often non-specific. The contrast enhancement on post-contrast T1-weighted images is established to be insufficient for the reliable determination of treatment response [2, 3], as it only provides details pertaining to contrast leakage from damaged vessels. Measures based on the non-enhancing region shown by increased FLAIR signals are suggested to be incorporated into assessment. This method, however, still has limitations in differentiating infiltrative tumors from other cause of increased signals, such as radiation effects [2].

Advanced MRI confers physiological information and may compensate for the non-specificity of structural imaging. Dynamic susceptibility contrast (DSC) is one of the most commonly-used perfusion techniques. Several biomarkers, including the relative cerebral blood volume (rCBV), mean transit time (MTT), and relative cerebral blood flow (rCBF), are calculated from the kinetics curve of contrast agent passing through the capillary bed [4]. DTI describes tumor microstructure by detecting water molecule mobility [5]. A decomposition into isotropic (p) and anisotropic (q) components from DTI was previously proposed [6], and showed utility in predicting tumor progression [7] and patient survival [8]. MR spectroscopy is an important method that detects metabolites and demonstrates significance in assessing tumor histology subtypes, malignancy grades, and treatment response [9, 10].

A series of quantitative imaging features can be extracted from MRI. In particular, histogram features can characterize tumor heterogeneity by measuring voxel distribution within tumor, and were related to tumor malignancy and patient survival [3]. As emerging advanced MRI modalities are developed to reflect tumor physiology, increasing numbers of features are generated. It remains a challenge to effectively incorporate the physiological information to reflect the multi-faceted characteristics of tumor. Further, selecting optimal features for clinical decision making is crucial.

Although machine learning algorithms have been successful in stratifying patients [11], classical machine learning techniques may not be effective in integrating the complementary information that multi-parametric MRI confers, with all features merged at an early stage, which may lead to highly noisy patterns. Further, the unique advantages from each individual feature view may be lost with data early integrated. The multi-view approach is a data integration method that was initially developed to jointly analyze multiple genomic data, i.e., gene expression and copy number variation [12]. It also has been applied to brain connectivity images for neurodegeneration type clustering [13]. This approach offers the advantage of parallelized feature selection from each individual view, and synthesizes the complementary information at a late stage. By doing so, it can avoid representation bias, since the analyses on each view are independent and integrated for final clustering [12].

As multi-parametric imaging may describe complementary information and because including multiple tumor regions may have additional value, here we hypothesized that the multi-view approach may be applied to improve tumor characterization through the fusion of multi-parametric MRI [13, 14]. Therefore, the purpose of this study was to determine whether the multi-view approach can effectively integrate histogram features of multi-parametric MRI for patient clustering, and whether the selected features can offer incremental values in survival prediction. The clinical characteristics and MR spectroscopy profiles of the identified patient clusters were compared.

## Material and methods

### Patients

From July 2010 to August 2015, suspected patients with supratentorial newly diagnosed glioblastoma were prospectively recruited. Patients were required to have good performance status (World Health Organization performance status 0-1) before surgery. Patients were excluded when they had the history of previous brain tumor, cranial surgery, radiotherapy/chemotherapy, or contraindication for MRI scan. This study was approved by the local institutional review board. Signed informed consent was obtained from all patients.

### Treatments

Neuronavigation and 5-aminolevulinic acid fluorescence were used to guide surgery, with other adjuvants (e.g., cortical and subcortical mapping, awake surgery, and intraoperative electrophysiology, when appropriate) to allow maximal safe resection. Extent of resection was assessed according to postoperative MRI within 72 h, classified as complete or partial resection of enhancing tumor or open biopsy [15]. Patient postoperative treatment was determined by the multi-disciplinary team in each case according to their postoperative status. All clinical, radiological, and histological data were collected prospectively. Clinical and radiological data were incorporated to evaluate response according to the Response Assessment in Neuro-oncology criteria [2]. Specifically, within the first 12 weeks after the completion of radiotherapy, progression was only determined if new enhancement was predominantly outside of the radiation field, unless pathological evidence was available. When patients were suspected of having pseudoprogression, our multi-disciplinary team continued current treatment, with close observation, until new evidence of true progression was confirmed. Therefore, for these patients, the progression-free survival was determined retrospectively.

### Preoperative MRI acquisition

MRI sequences were acquired on a 3-Tesla scanner (Magnetron Trio; Siemens Healthcare, Erlangen, Germany) with a standard 12-channel receive-head coil. MRI sequences included post-contrast T1W, T2W, T2W-FLAIR, DSC, DTI, and multi-voxel 2D ^1^H-MRS chemical shift imaging (CSI). PRESS excitation was selected to encompass a grid of 8 rows × 8 columns on T2W images. The scanning details are in Supplementary Methods.

### Imaging processing

All images were co-registered to T2W images, where the CSI was planned. The image co-registration was performed using the linear registration tool (FLIRT) in Oxford Centre for Functional MRI of the Brain Software Library (FSL) v5.0.0 (Oxford, UK) [16, 17]. DSC was processed and leakage correction was performed using NordicICE (NordicNeuroLab). The arterial input function was automatically defined. The rCBV, MTT, and rCBF maps were calculated. DTI was processed using the diffusion toolbox in FSL [18]. Normalization and eddy current correction were performed. The isotropic (p) and anisotropic (q) components were calculated [6].

### Regions of interest

Tumor ROIs were manually delineated on the post-contrast T1W and FLAIR images using 3D slicer v4.6.2 (https://www.slicer.org/) [19]. The delineation was independently performed by a neurosurgeon (CL, > 8 years of experience), and a researcher (NRB, > 4 years of image analysis experience), and reviewed by a neuroradiologist (TM, > 8 years of experience). Each author used consistent criteria in each patient, and was blinded to the patient clustering and outcomes. Non-enhancing (NE) ROIs were defined as the non-enhancing regions outside of contrast-enhancing (CE) regions, obtained by a Boolean subtraction of CE and FLAIR ROIs in MATLAB (version 2016a, MathWorks, Inc.). For each subject, regions of normal-appearing white matter were manually segmented in contralateral white matter and used as normal controls [20]. This region was typically located in the white matter which was furthest in distance from the tumor location and has no perceivable abnormalities. Each tumor voxel was normalized by dividing it by the mean value of normal-appearing white matter. Inter-rater reliability was performed using Dice similarity coefficient scores.

### Histogram features

**Fig. 1:**
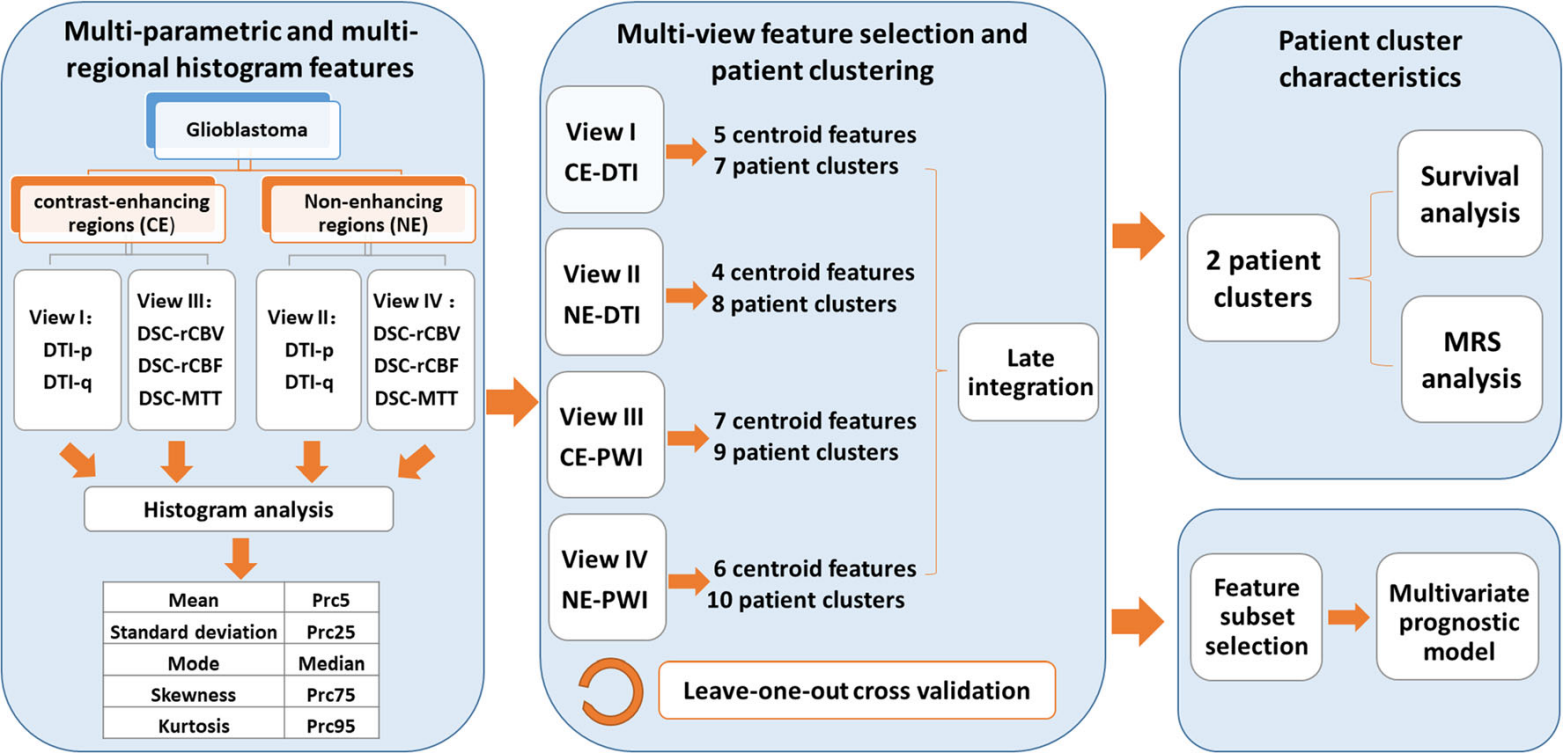
Study design. DTI-p and DTI-q maps are generated from diffusion tensor imaging (DTI). The relative cerebral blood volume (rCBV), mean transit time (MTT), and relative cerebral blood flow (rCBF) maps are generated from dynamic susceptibility contrast (DSC) imaging. Histogram features extracted from the multiple modalities and regions (contrast-enhancing and non-enhancing) are treated as four independent views. Each view is firstly clustered to select centroid features, which are later used to cluster patients. The resulting clusters from each view are integrated to yield two final patient clusters. A leave-one-out cross validation is performed. Patient clusters are assessed in survival analysis and their metabolic signatures are compared. The centroid features are ranked according to the importance in the clustering and selected features are used to build multi-variate prognostic model

The study design is summarized in Fig. 1. Histogram features were extracted using MATLAB. Perfusion images (rCBV, MTT, and rCBF) and diffusion images (DTI-p and DTI-q) were analyzed separately. The CE and NE ROIs were also analyzed independently. Therefore, four categories of feature sets (CE-diffusion, NE-diffusion, CE-perfusion, NE-perfusion) were obtained. Intensity histograms were constructed using 100 bins. As shown in Fig. 1, a total of 10 histogram features were calculated, including mean, standard deviation (SD), median, mode, skewness, kurtosis, and 5th (Prc5), 25th (Prc25), 75th (Prc75), and 95th (Prc95) percentiles. Therefore, altogether 100 features were extracted from each subject.

### Multi-view feature selection and clustering

The analysis was performed using a multi-view late integration methodology called multi-view biological data analysis (MVDA), implemented in R and available from GitHub (https://github.com/angy89/MVDA). Late integration methodologies allow analyzing each view independently and then merging the results [21].

The analysis was divided into multiple steps [12]. Briefly, the features were firstly clustered using the hierarchical ward clustering method in each view. The number of features was reduced by selecting the centroids of feature clusters, according to the correlation within each feature cluster. Next, for each view, the patients were clustered by applying a hierarchical ward clustering method using the features selected from last step. Lastly, the clustering results of each view were integrated in a late integration method to yield two final patient clusters. Analysis details are in Supplementary Methods.

### Leave-one-out cross validation

To validate patient clustering was not obtained by random, a leave-one-out cross-validation (LOOCV) procedure was applied. Briefly, all steps of MVDA approach were repeated by leaving one patient out at each repetition. The consensus analysis was performed in the 80 clustering results obtained from the LOOCV approach. An 80 × 80 co-occurrence consensus clustering matrix M was created, where M (*i*, *j*) indicating percentage of times that the patients *i* and *j* were clustered together across the 80 dataset perturbations.

### Multi-voxel MRS processing

CSI data were processed using LCModel (Provencher). Choline (Cho) and N-acetylaspartate/creatine (NAA) were calculated as a ratio to creatine (Cr). All relevant spectra from CSI voxels were assessed for artifacts using described criteria [22]. The values of the Cramer-Rao lower bounds were used to evaluate the quality and reliability of CSI data. CSI values with SD > 20% were discarded. The DTI and PWI images were co-registered to T2-space, which was used to plan CSI acquisition. To account for the resolution difference between T2- and CSI space, all co-registered data were projected to CSI space, according to their coordinates using MATLAB. The proportion of T2-space tumor pixels occupying each CSI voxel was calculated. Only CSI voxels containing more than 50% tumor T2-voxels were included for further analysis.

### Feature ranking

To estimate the contribution of each centroid feature in the clustering, the variable importance evaluation function “varImp” in the R package “Caret” was used [23]. The patient clustering result was firstly used to train a logistic regression model, which was used to evaluate the importance of each feature, according to the model performance. The feature importance was scored and scaled with a maximum value of 100.

### Statistical analysis

All analyses were performed in RStudio v3.2.3. CSI data were compared with Wilcoxon rank sum test using Benjamini-Hochberg procedure for controlling the false discovery rate in multiple comparisons. Kaplan-Meier and Cox proportional hazards regression analyses were performed to evaluate patient survival. For Cox proportional hazards regression, all relevant covariates, including IDH-1 mutation and MGMT methylation status, sex, age, extent of resection, and contrast-enhancing volume were considered. For Kaplan-Meier analysis using log-rank test, each feature was dichotomized using optimal cutoff values calculated by “surv_cutpoint” function in the R Package “survminer.” Patients alive at the last known follow-up were censored. Logistic regression was used to test prognostic values of covariates for 12-month overall survival (OS) and progression-free survival (PFS). The baseline models were constructed using all relevant clinical covariates. Histogram features were subsequently added into baseline models. The incremental prognostic values of the models with histogram features were determined by comparing AUC using one-way ANOVA. The hypothesis was accepted at a two-sided significance level of alpha = 0.05.

## Results

### Patients and regions of interest

A total of 136 patients were recruited for MRI scan and surgery. Twenty-one patients were excluded due to non-glioblastoma pathology diagnosis after surgery. Postoperatively, patients received concurrent temozolomide chemoradiotherapy followed by adjuvant temozolomide following the Stupp protocol (73.0%, 84), short-course radiotherapy (17.4%, 20/115), or best supportive care (9.6%, 11/115), respectively. Among the 84 patients, four patients were lost in follow-up and excluded. A total of 80 patients were finally included into the study. Characteristics of 80 patients and two patient clusters were summarized in Table 1.

**Table 1:** Clinical characteristics

Variable	Patient number			*p* value
	Total (*n* = 80)	Cluster 1 (*n* = 53)	Cluster 2 (*n* = 27)	
Age at diagnosis				
< 60	35	18	16	0.058
≥ 60	45	35	11	
Sex				
Male	58	41	17	0.201
Female	22	12	10	
Extent of resection (of enhancing tumor)				
Complete resection	56	35	21	0.267
Partial resection	22	17	5	
Biopsy	2	1	1	
MGMT-methylation status*				
Methylated	37	24	13	0.929
Unmethylated	41	27	14	
IDH-1 mutation status				
Mutant	7	4	3	0.622
Wild-type	73	49	24	
Preoperative tumor volumes (cm^3^) ^#^				
Contrast-enhancing	49.7 ± 28.1	50.2 ± 28.4	50.4 ± 28.1	0.823
Non-enhancing	64.7 ± 48.3	48.7 ± 27.9	92.8 ± 53.5	*0.007*
Survival (days)				
Median OS (range)	461 (52–1259)	424 (52–839)	689 (109–1259)	*0.020* ^†^
Median PFS (range)	264(25–1130)	248 (25–607)	318 (279–1130)	*< 0.001* ^†^

Italics: *p* < 0.05

*MGMT-methylation status unavailable for 2 patients; ^**#**^mean ± SD of original data; ^†^log-rank test

*MGMT* O-6-methylguanine-DNA methyltransferase, *IDH-1* isocitrate dehydrogenase 1, *OS* overall survival, *PFS* progression-free survival

Inter-rater reliability testing of ROIs showed fair agreement between the raters, with Dice scores 0.85 ± 0.10 (mean ± SD) for CE and 0.86 ± 0.10 of FLAIR ROIs respectively.

### Identification of patient clusters

**Fig. 2:**
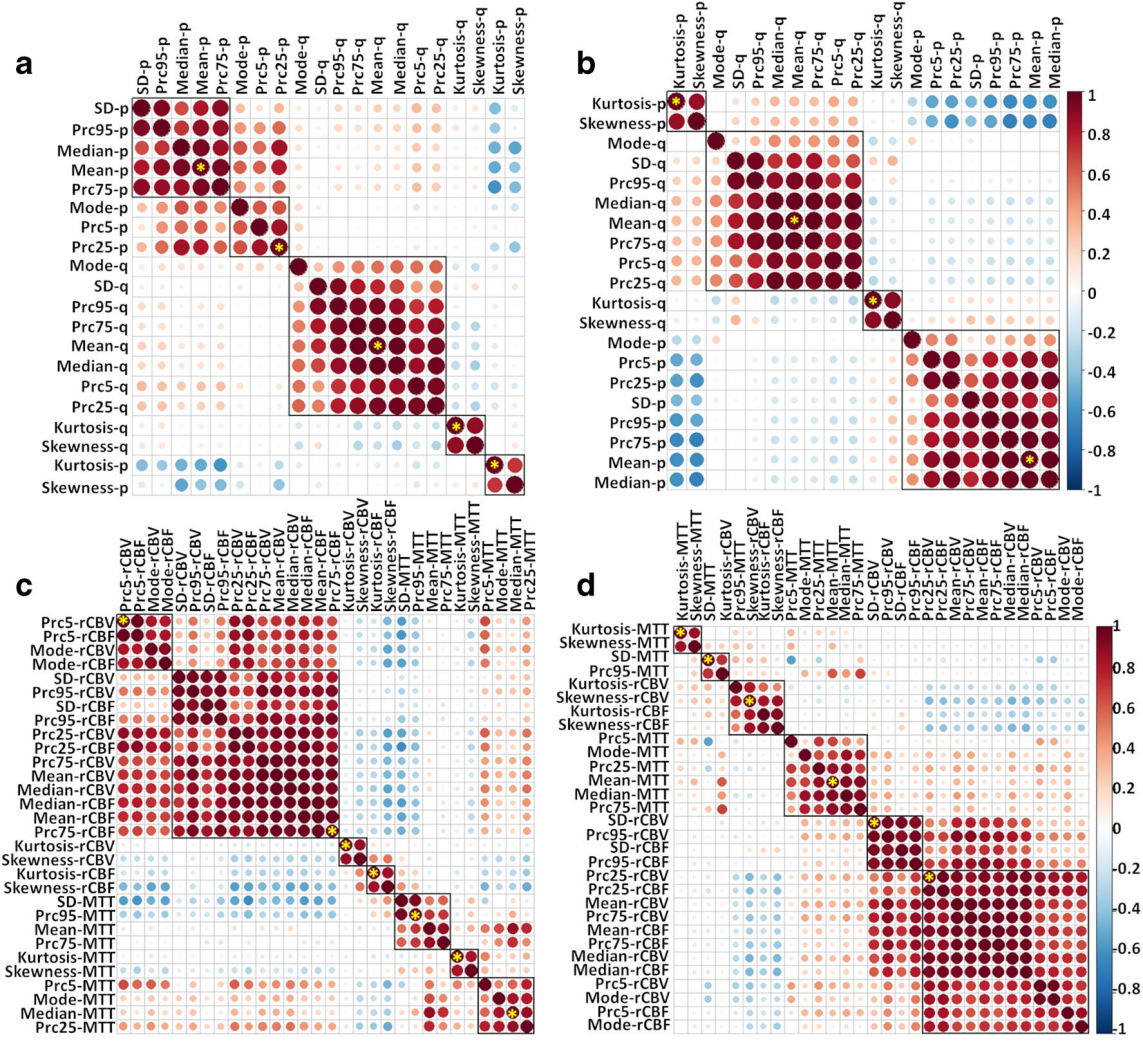
Multi-view feature selection. In each view, all features are clustered using the hierarchical ward clustering method. The centroid features (marked by yellow stars) are selected to represent each view. **a** View 1 (CE-DTI); **b** view 2 (NE-DTI); **c** view 3 (CE-PWI); **d** view 4 (NE-PWI)

From the four views, 5, 4, 7, and 6 centroid features were respectively selected (Fig. 2, Table 2). Using the centroid features and optimal cluster numbers determined in the algorithm, patients were firstly divided into 7, 8, 9, and 10 clusters in each view, using the hierarchical ward clustering. Late integration of four views yielded a final clustering of two patient clusters, with 53 and 27 patients in each cluster respectively.

**Table 2:** Centroid features in each view

View	Features
View1: CE-diffusion	Mean-p-CE
	Prc25-p-CE
	Kurtosis -p-CE
	Mean-q-CE
	Kurtosis-q-CE
View2: NE-diffusion	Mean-p-NE
	Kurtosis-p-NE
	Mean-q-NE
	Kurtosis-q-NE
View3: CE-perfusion	Prc75-rCBF-CE
	Prc5-rCBV-CE
	Kurtosis-rCBV-CE
	Kurtosis-rCBF-CE
	Prc95-MTT-CE
	Median-MTT-CE
	Kurtosis-MTT-CE
View4: NE-perfusion	Prc25-rCBV-NE
	SD-rCBV-NE
	Skewness-rCBV-NE
	Median-MTT-NE
	SD-MTT-NE
	Kurtosis-MTT-NE

*CE* contrast-enhancing region, *NE* non-enhancing region, *Prc25/Prc75/Prc95* 25th/75th/95th percentiles of histogram

### Leave-one-out cross validation of patient clustering

**Fig. 3:**
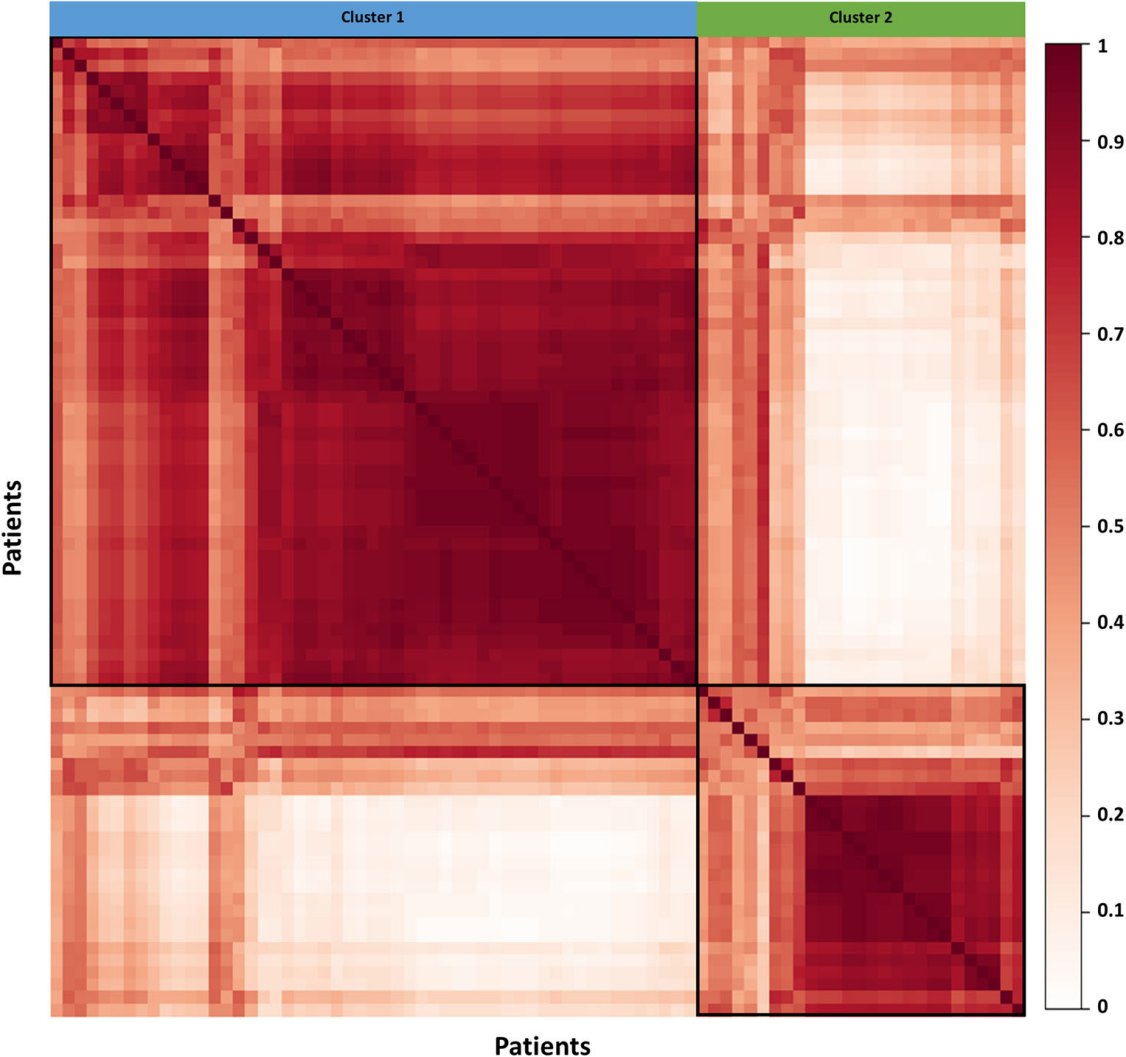
Leave-one-out cross validation of patient clustering. After multi-view clustering, consensus analysis was performed based on the 80 clustering results obtained after the leave-one-out cross validation. The mean value of the co-occurrence consensus clustering matrix was 0.79 for patient Cluster 1 and 0.68 for patient Cluster 2

After leave-one-out cross validation, the co-occurrence consensus clustering matrix was computed. The result showed that the two patient clusters generated from the unsupervised clustering were stable. The mean values of the co-occurrence consensus clustering matrix were 0.79 for Cluster 1 and 0.68 for Cluster 2 (Fig. 3).

### Clinical relevance of patient clusters

**Fig. 4:**
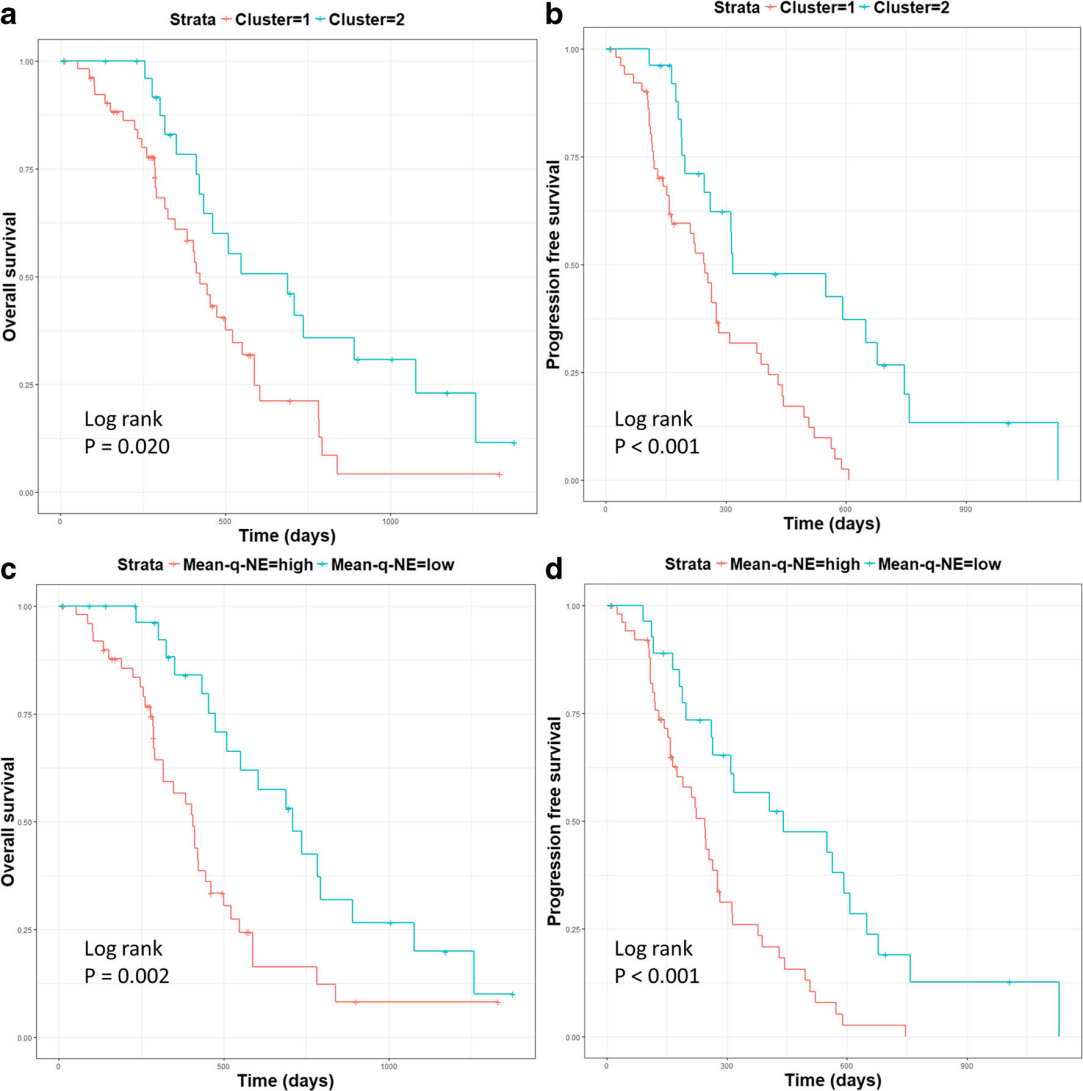
Survivals of patient clusters. Log-rank test showed patient Cluster 2 displayed better OS (*p* = 0.020) (**a**) and PFS (*p* < 0.001) (**b**). Higher man value of DTI-q in the non-enhancing region (Mean-q-NE) was associated with a worse OS (*p* = 0.002) (**c**) and PFS (*p* < 0.001) (**d**)

The two patient clusters showed no significant differences in clinical characteristics (Table 1). Interestingly, two clusters had similar contrast-enhancing tumor volume (*p* = 0.823). Cluster 1, however, had significantly smaller non-enhancing tumor volume (*p* = 0.007) than Cluster 2. Further, the two clusters showed significant difference in survival. Specifically, Cluster 2 showed better OS (log-rank test, *p* = 0.020) and better PFS (log-rank test, *p* < 0.001) than Cluster 1 in Kaplan-Meier analysis (Table 1, Fig. 4a and b).

Since MGMT promoter methylation status was missing in two patients, the multi-variate Cox proportional hazards regression modeling was tested in the remaining 78 patients. The results showed that Cluster 2 displayed significantly better OS (*p* = 0.007, HR = 0.32) and PFS (*p* < 0.001, HR = 0.33) than Cluster 1, considering relevant covariates. Among these covariates, extent of resection (*p* = 0.019, HR = 2.20) and contrast-enhancing tumor volume (*p* < 0.001, HR = 1.02) significantly affected OS. Extent of resection (*p* = 0.003, HR = 2.84) significantly affected PFS. No significance was found in other clinical factors.

### Metabolic signatures of patient clusters

Due to the abovementioned rules excluding CSI voxels containing less than 50% tumor, CSI data were missing in four patients. Our results showed NAA/Cr ratio in NE region of Cluster 2 was significantly lower than Cluster 1 (*p* = 0.040) after controlling multiple comparisons. The comparison of CSI data in two patient clusters are detailed in Supplementary Table S1.

### Feature ranking and prognostic performance of features

**Fig. 5:**
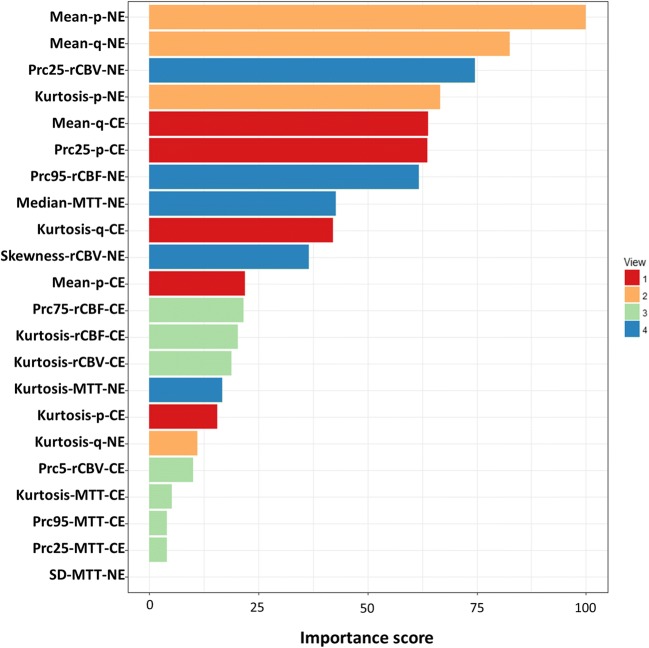
Feature ranking. The centroid features were ranked according to the importance in the clustering. The scores were scaled with a maximum value of 100

Seven features with a score over 50 were selected, according to the importance of centroid features in the clustering (Fig. 5). All the seven features showed significance in survival analysis (Table 3). Particularly, higher Mean-q-NE was associated with worse OS (HR = 1.40, *p* = 0.020) and worse PFS (HR = 1.36, *p* = 0.031). The Kaplan-Meier curves showing the relevance of Mean-q-NE in OS and PFS are demonstrated by Fig. 4c and d.

**Table 3:** Survival statistics of selected feature

Feature	Progression-free survival*				Overall survival*			
	HR	95% CI	*p* value (Cox regression)	*p* value (log-rank)	HR	95% CI	*p* value (Cox regression)	*p* value (log-rank)
Mean-p-NE	0.79	0.58–1.08	0.143	*< 0.001*	0.74	0.53–1.04	0.083	*0.015*
Mean-q-NE	1.40	1.05–1.86	*0.020*	*< 0.001*	1.36	1.03–1.79	*0.031*	*0.002*
Prc25-rCBV-NE	1.28	0.94–1.74	0.121	0.052	1.53	1.09–2.14	*0.014*	*0.019*
Kurtosis-p-NE	1.18	0.85–1.63	0.326	0.168	1.66	1.15–2.39	*0.007*	*0.048*
Mean-q-CE	1.17	0.90–1.51	0.245	*0.029*	1.17	0.89–1.55	0.268	0.197
Prc25-p-CE	0.88	0.66–1.17	0.369	*0.032*	0.79	0.56–1.10	0.165	*0.004*
Prc95-rCBF-NE	1.11	0.88–1.40	0.358	*0.049*	1.15	0.88–1.51	0.307	0.063

Italics: *p* < 0.05

*Cox models accounted for IDH-1 mutation status, MGMT methylation status, sex, age, extent of resection, and contrast-enhancing tumor volume

*HR* hazard ratio, *CI* confidence interval, *p* isotropic diffusivity of DTI, *q* anisotropic diffusivity of DTI, *Prc25/Prc95* 25th/95th percentiles of histogram, *CE* contrast-enhancing region, *NE* non-enhancing region

**Fig. 6:**
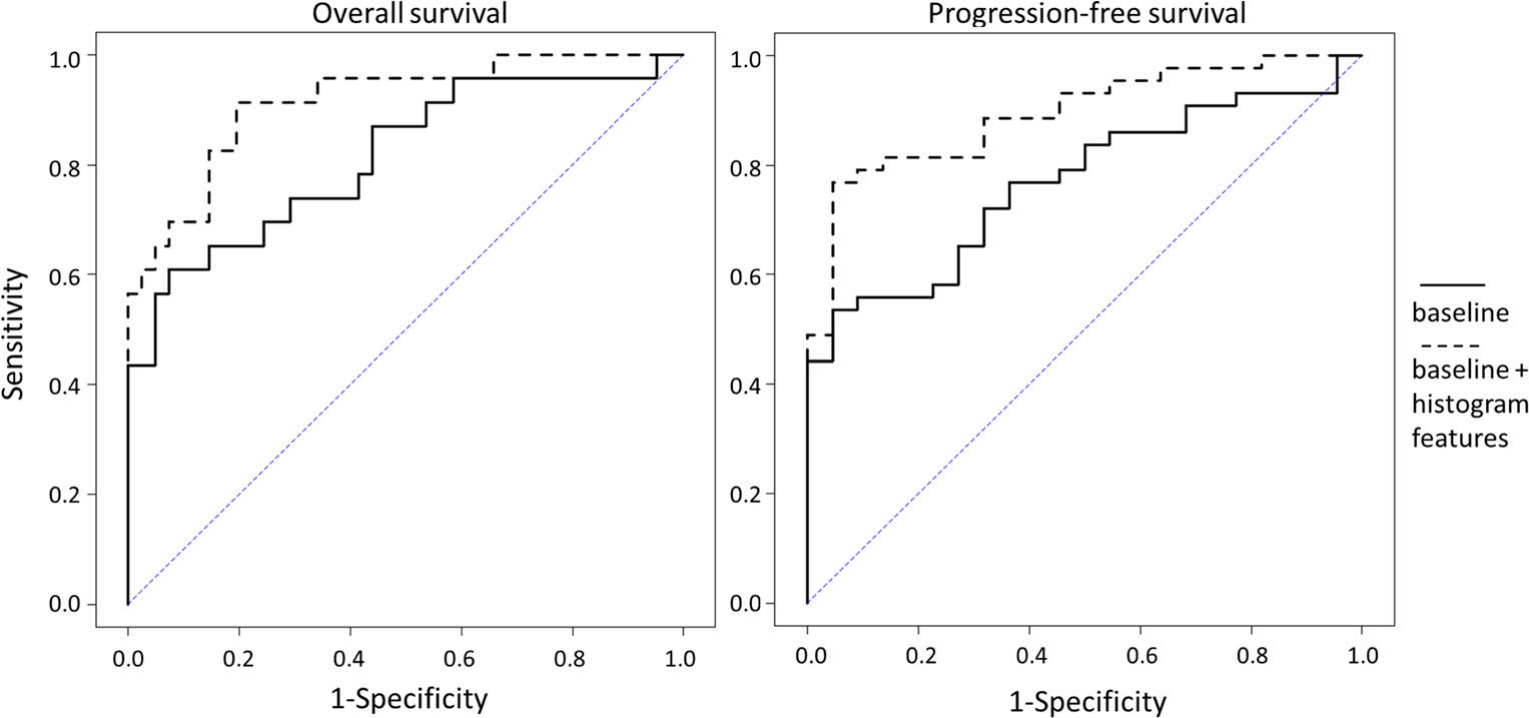
ROC curve analysis. ROC curves showed that the models of 12-month OS (left) and PFS (right) were significantly improved (*p* = 0.020, and *p* = 0.022 respectively) by adding the seven most important histogram features into the baseline models

For prediction of 12-month OS and PFS, the AUC of baseline multi-variate models were 0.81 (95% CI, 0.70–0.93) and 0.77 (95% CI, 0.65–0.88) respectively. The results of model comparison showed that the seven features significantly improved both OS model (AUC, 0.91; 95% CI, 0.84–0.99; *p* = 0.020) and PFS model (AUC, 0.89; 95% CI, 0.81–0.97; *p* = 0.022) (Fig. 6).

## Discussion

This study showed that integrating multi-parametric and multi-regional MRI histogram features may help to identify tumor phenotypes correlating patient prognosis, using a multi-view approach. The histogram features from advanced MRI showed incremental prognostic value over clinical variables.

Evidences support the utility of histogram features from advanced MRI in patient stratification and survival prediction [24–26]. Limited studies, however, have investigated both perfusion and diffusion imaging parameters simultaneously [27–29]. Further, as perfusion and diffusion imaging reflect different physiological facets, we hypothesized that integrating them effectively may lead to a better tumor characterization. With this unsupervised algorithm, we separated patients into two clusters with distinct survivals and metabolic signatures, suggesting the importance of appropriately integrating multiple modalities for tumor characterization. One recent study, however, showed that diffusion and perfusion histogram parameters showed marginal values [27]. The different findings may result from the differences in selection of imaging markers and features, and patient treatment strategies. Future studies using prospective design are needed to further validate the prognostic value of these imaging parameters.

The non-enhancing peritumoral areas visualized on FLAIR images may include both infiltrative tumor regions and edematous brain parenchyma, and thus may not be specific in indicating tumor aggressiveness. Several recent studies showed that pre-treatment FLAIR volume is not predictive of patient survival, whereas the increased volume on FLAIR during adjuvant therapy was associated with worsened survival [30–32]. The above findings may imply that a better characterization of pre-treatment non-enhancing region using advanced MRI may have potential to extract clinically relevant information, which contributes to our motivation for separating non-enhancing regions from contrast-enhancing regions.

Our results showed higher Mean-q-NE (mean value of DTI-q in NE region) was associated with worse survivals. Correspondingly, MRS showed that the worse survival group had higher NAA/Cr in NE region. Glioblastoma is recognized to preferentially migrate along white matter tracts, which may lead to increased anisotropic diffusivity. NAA is a marker of neuronal integrity [9], while DTI-q indicates anisotropic diffusivity [33]. Previous studies showed that an increased anisotropic diffusivity and decreased isotropic diffusivity in the non-enhancing tumor region could indicate tumor infiltration in this area and was associated with tumor progression [20]. A retrospective study revealed that more extensive resection of q abnormalities was associated with better overall survival [34]. Our results suggest that the worse survival group may have more intact neurons that may facilitate tumor infiltration. Since this cohort has received maximal safe surgery aiming to resect CE region, the infiltration in NE region thus would be more responsible for treatment failure.

Radiomics approach can extract high-throughput features from medical images [35, 36]. The increasing number of features, however, may pose the challenges of effective feature selection and modality integration for robust phenotyping [37, 38]. Currently, many techniques have been developed for this purpose [39]. To maximize the specific tumor biology information conferred by physiological imaging, new approaches with biological hypothesis might be needed to characterize the complex tumor. Considering the interpretability and robustness of features, our current study investigated the feasibility of applying a genomic tool to histogram features. Future work may be extended to broader imaging feature sets and bioinformatics tools.

Our study has limitations. Firstly, although we used a leave-one-out cross validation, the patient population reported is from a single center. The purpose of this study however is to introduce a method that could potentially integrate multi-parametric MRI. Future studies aim to validate the findings of this study using a multi-center cohort. Secondly, although previous studies have validated the histological correlates of the imaging markers, our current findings need further biological validation. Lastly, as the ^1^H-MRS voxels were larger than T2 space voxels, we had fewer patients with CSI data available.

In conclusion, our results showed that the multi-view clustering method can provide an effective approach of integrating multiple quantitative MRI features. The histogram features selected from the proposed approach may be used as potential prognostic imaging markers.

## Electronic supplementary material

ESM 1 (DOCX 52 kb)

## Abbreviations

CE: Contrast-enhancing
CSI: Chemical shift imaging
DTI-p: Isotropic diffusivity
DTI-q: Anisotropic diffusivity
LOOCV: Leave-one-out cross-validation
MVDA: Multi-view biological data analysis
NE: Non-enhancing
OS: Overall survival
PFS: Progression-free survival
