# Supplementary material for: Multi-parametric and multi-regional histogram analysis of MRI: modality integration reveals imaging phenotypes of glioblastoma
Source: Eur Radiol. 2019 Feb 1;29(9):4718–29. doi: 10.1007/s00330-018-5984-z (PMC6682853; doi:10.1007/s00330-018-5984-z)
Supplement: Supplementary file 1 — (DOCX 52 kb) [file 330_2018_5984_MOESM1_ESM.docx]

**Supplementary Methods**

**MRI protocols**

Post-contrast T1W (TR/TE/TI 2300/2.98/900 ms; flip angle 9°; FOV 256 × 240 mm; 176-208 slices; no slice gap; voxel size 1.0 × 1.0 × 1.0 mm) after intravenous injection of 9 mL gadobutrol (Gadovist,1.0 mmol/mL; Bayer); T2W (TR/TE 4840-5470/114 ms; refocusing pulse flip angle 150°; FOV 220 × 165 mm; 23-26 slices; 0.5 mm slice gap; voxel size of 0.7 × 0.7 × 5.0 mm); T2W-FLAIR (TR/TE/TI 7840-8420/95/2500 ms; refocusing pulse flip angle 150°; FOV 250 × 200 mm; 27 slices; 1 mm slice gap; voxel size of 0.78125 × 0.78125 × 4.0 mm). Perfusion imaging was acquired with a DSC sequence by employing a single-shot echo planar imaging (EPI) technique (TR/TE 1500/30 ms; flip angle 90°; FOV 192 × 192 mm; 19 slices; slice gap 1.5 mm; voxel size of 2.0 × 2.0 × 5.0 mm;) with 9 mL gadobutrol (Gadovist 1.0 mmol/mL) followed by a 20 mL saline flush administered via a power injector at 5 mL/s. DTI was acquired before contrast imaging, using a single-shot echo-planar sequence (TR/TE 8300/98 ms; flip angle 90°; FOV 192 × 192 mm; 63 slices; no slice gap; voxel size 2.0 × 2.0 × 2.0 mm; 12 directions; b values: 350, 650, 1000, 1300, and 1600 sec/mm^2^; imaging time: 9 minutes 26 seconds). Multivoxel 2D ^1^H-MRS utilized a semi-LASER sequence (TR/TE 2000/30-35 ms; flip angle 90°; FOV 160 × 160 mm; voxel size 10 × 10 × 15-20 mm). PRESS excitation was selected to encompass a grid of 8 rows × 8 columns on T2W images.

**Multi-view feature selection and clustering**

The analysis was divided into multiple steps: **I**. To reduce the dimensionality and remove noisy information, the features were first clustered using the hierarchical ward clustering method for each view. The number of feature clusters was determined by the previously proposed VAL index [1]. Clustering solutions with high correlation within each cluster and low correlation between the clusters were preferred. The number of features was reduced by selecting the centroids of the feature clusters which represent the features of each view. **II**. For each view, the patients were clustered by applying a hierarchical ward clustering method using the features selected from the previous step. The number of patient clusters was also determined by the VAL index [1]. **III**. The clustering results of each view were integrated in a late integration method. The vector of clustering assignment of each view was transformed into a binary membership matrix, with patients on the rows and clustering on the columns. These matrices were transposed and stacked vertically to create a larger matrix X with L rows (the clusters) and N columns (the patients). This matrix was then factorized to obtain two matrices P (with L rows and k columns) and H (with k rows and N columns), minimizing the difference between X and PH. In this settings H represented the membership matrices of the N patients to the final multi-view clusters. The number of multi-view clusters was set to 2 to dichotomize patients into two final clusters with better or worse survivals respectively.

1 Serra A, Fratello M, Fortino V, Raiconi G, Tagliaferri R, Greco D (2015) MVDA: a multi-view genomic data integration methodology. Bmc Bioinformatics 16


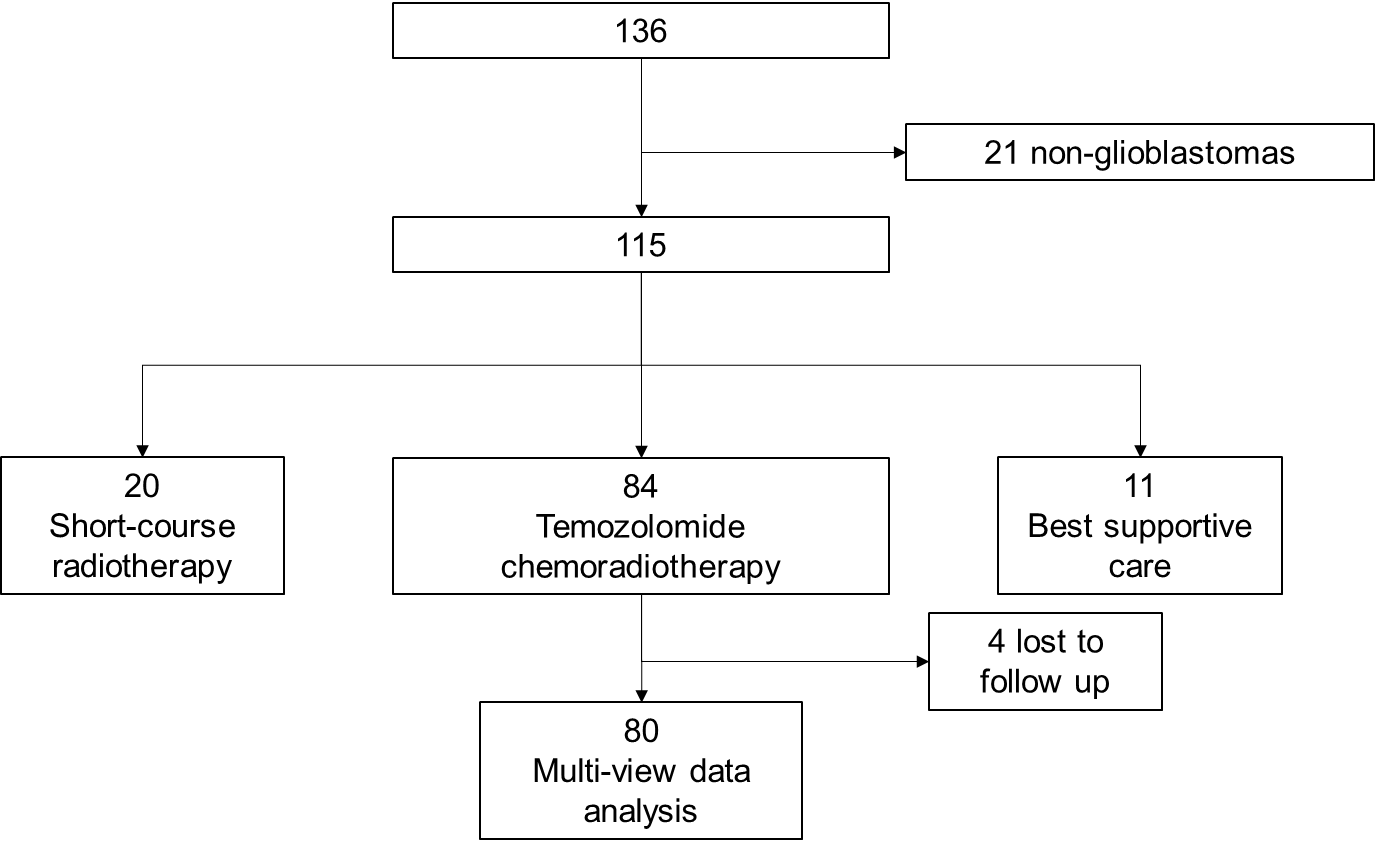


**Supplementary Figure S1. Flowchart showing how patients were excluded.** A total of 136 patients with suspected diagnosis of glioblastoma were prospectively recruited and underwent surgery. Twenty-one patients were excluded according to the post-operative pathology. The excluded pathologies include oliogoastrocytoma, oligodendroglioma, anaplastic astrocytoma, anaplastic oliogoastrocytoma, anaplastic ependymoma, anaplastic neurocytoma, and atypical meningioma. Among the 115 included patients, 84 patients received concurrent temozolomide (TMZ) chemoradiotherapy followed by adjuvant TMZ after surgery. Four patients were lost to follow up. Eighty patients were included for the multi-view data analysis.

| **Supplementary Table S1. Metabolic statistics of patient clusters** | | | | | | |
| --- | --- | --- | --- | --- | --- | --- |
| **Contrast-enhancing tumor region** | | | | | | |
| Metabolite | Cluster 1 | | Cluster 2 | | | *P* value |
|  | Mean ± SD | 95% CI | Mean ± SD | 95% CI | |  |
| Cho/Cr | 0.70 ± 0.24 | 0.63-0.77 | 0.63 ± 0.17 | 0.55-0.70 | | 0.715 |
| NAA/Cr | 0.76 ± 0.35 | 0.66-0.86 | 0.46 ± 0.27 | 0.35-0.58 | | 1.000 |
| Cho/NAA | 0.60 ± 0.32 | 0.50-0.69 | 0.49 ± 0.26 | 0.38-0.60 | | 0.448 |
| **Non-enhancing tumor region** | | | | | | |
| Metabolite | Cluster 1 | | Cluster 2 | | *P* value | |
|  | Mean ± SD | 95% CI | Mean ± SD | 95% CI |  |  |
| Cho/Cr | 0.44 ± 0.13 | 0.40-0.48 | 0.40 ± 0.14 | 0.34-0.46 | 0.149 | |
| NAA/Cr | 0.97 ± 0.32 | 0.86-1.07 | 0.76 ± 0.30 | 0.64-0.89 | **0.040** | |
| Cho/NAA | 0.54 ± 0.50 | 0.38-0.70 | 0.45 ± 0.19 | 0.37-0.53 | 0.689 | |
| Cho: Choline; NAA: N-acetyl aspartate; Cr: creatine. | | | | | | |
